# Supplementary material for: Understanding the Causes of Frailty Using a Life-Course Perspective: A Systematic Review
Source: Healthcare (Basel). 2023 Dec 21;12(1):22. doi: 10.3390/healthcare12010022 (PMC10778671; doi:10.3390/healthcare12010022)
Supplement: Supplementary file 1 [file healthcare-12-00022-s001.zip › healthcare-2773429-supplementary.pdf]

Table S1. Search strategy of the systematic review of cohort studies examining associations of risk factors with frailty

“frail∗.mp.” OR “accumulated deficit.mp.” OR “cumulative deficit.mp.” AND "risk factors.mp." OR "predictive factors.mp." AND “Cohort.mp.” OR “Prospective.mp.” OR “Life-course.mp.”

Table S2. Physical activity and nutritional variables as covariates in 33 studies examining the association between risk factors and frailty

**Studies**	**Covariates** Physical activity	Energy Intake	Quality Eating Indexes	Fruits and Vegetables	SSBs	Red meat
Millar et al. 2022	None	Adjusted	None	None	None	None
Strandberg et al. 2018	Adjusted	None	None	None	None	None
Susanto et al. 2018	Adjusted	None	None	None	None	None
Sotos-Prieto et al. 2022	None	Adjusted	None	None	None	None
Landré et al. 2020	None	None	None	None	None	None
Baranyi et al. 2022	None	None	None	None	None	None
Strandberg et al. 2012	None	None	None	None	None	None
Kheifets et al. 2022	None	None	None	None	None	None
Landré et al. 2020	None	None	None	None	None	None
Wennberg et al. 2021	None	None	None	None	None	None
Bouillon et al. 2013	None	None	None	None	None	None
Pilleron et al. 2016	None	None	None	None	None	None
Haapanen et al. 2018a	None	None	None	None	None	None
Haapaanen et al. 2018b	None	None	None	None	None	None
Fung et al. 2020	Adjusted	Adjusted	Adjusted	None	None	None
Gil-Salcedo et al. 2020	Adjusted	None	None	Adjusted	None	None
Haapaanen et al. 2018c	None	None	None	None	None	None
Orkaby et al. 2022	None	None	None	None	None	None
Savela et al. 2013	None	None	None	None	None	None
Li et al. 2020	None	None	None	None	None	None
Yeung et al. 2020	Adjusted	None	None	None	None	None
Brunner et al. 2018	None	None	None	None	None	None
Stenhold et al. 2013	Adjusted	None	None	None	None	None
Walker et al. 2018	None	None	None	None	None	None
Sodhi et al. 2019	None	None	None	None	None	None
Struijk et al. 2022	Adjusted	Adjusted	None	Adjusted	Adjusted	Adjusted
Dugravot et al. 2019	None	None	None	None	None	None
Hoogendijk et al. 2017	None	None	None	None	None	None
Yu et al. 2020	Adjusted	None	None	None	None	None
Struijk et al. 2020	Adjusted	Adjusted	Adjusted	None	None	None
Landré et al. 2023	Adjusted	None	None	Adjusted	None	None
Niederstrasser et al. 2019	Adjusted	None	None	None	None	None
Amieva et al. 2022	None	None	None	None	None	None

Legend: None; Adjusted in the model

SSBs: Sugar Sweetened Beverages
